# The consequences of chaos: Foraging activity of a marine predator remains impacted several days after the end of a storm

**DOI:** 10.1371/journal.pone.0254269

**Published:** 2021-07-09

**Authors:** Emmanuelle Barreau, Akiko Kato, Andre Chiaradia, Yan Ropert-Coudert

**Affiliations:** 1 Centre d’Études Biologiques de Chizé, UMR 7372 CNRS—La Rochelle Université, Villiers en Bois, France; 2 Conservation Department, Phillip Island Nature Parks, Cowes, VIC, Australia; Hawaii Pacific University, UNITED STATES

## Abstract

As extreme weather is expected to become more frequent with global climate change, it is crucial to evaluate the capacity of species to respond to short-term and unpredictable events. Here, we examined the effect of a strong storm event during the chick-rearing stage of little penguins (*Eudyptula minor*) from a mega colony in southern Australia. We investigated how a 3-day storm affected the foraging behaviour of little penguins by comparing their foraging activities and body mass change before, during and after the storm event. As strong winds deepened the mixed layer in the birds’ foraging zone during the storm, penguins increased their foraging trip duration, had a lower prey encounter rate and a lower body mass gain. The adverse effects on the foraging efficiency of little penguins continued several days after the storm ceased; even though the water column stratification had returned as before the storm, suggesting a prolonged effect of the storm event on the prey availability. Thus, short-term stochastic events can have an extended impact on the foraging efficiency of penguins. When occurring at a crucial stage of breeding, this may affect breeding success.

## Introduction

Foraging in an open environment is challenging for marine species, as many oceanographic features and processes operate from broad to fine spatial and temporal scales, influencing the abundance and distribution of oceanic organisms. For instance, marine predators use oceanic fronts to forage better in areas where resources are most predictable [1, 2]. They can adjust their search pattern in response to changes in environmental conditions [1–4].

As inshore feeders and central place foragers at breeding, little penguins (*Eudyptula minor*) locate prey in a small foraging range and at short time scales to feed themselves and provide to their offspring [5]. Like many other marine predators [1, 3, 6], little penguins can enhance their foraging success when water masses are thermally stratified: the presence of a thermocline in the water column acts as a thermal barrier to their ectothermic preys, aggregating them in a narrow band in the water column where prey are more accessible to target [7, 8]. However, thermal stratification can vary in space and time within years or weeks in the foraging areas of these birds [7, 8]. In addition, one-off events, such as strong winds and storms, may increase the mixing of the water column, leading to the weakening or total disappearance of the thermocline [7] and, consequently, affecting little penguins’ foraging efficiency [9, 10].

Here, we report on the effect of a strong storm event (>6 the Beaufort scale, Australian Bureau of Meteorology) on the foraging efficiency and body mass of little penguins at Phillip Island during the 2018–19 breeding season. Phillip Island is located in the Bass Strait, Australia, where the marine system is experiencing rapid environmental changes [11, 12]. The storm event occurred during the chick-rearing period when both parents alternate one-day foraging trip at sea. We hypothesise that little penguins increase their foraging effort in response to the storm event that may have altered and disturbed the thermocline’s presence and depth. We examined how the penguins’ foraging behaviour was affected by changes in the water column thermal structure at a daily scale at three different stages: before, during and after the storm event.

## Material & methods

### Study site and data collection

Fieldwork was conducted at the Penguin Parade® little penguins breeding colony on Phillip Island, Victoria, Australia (38°31’ S, 145°09’ E) during the chick-guard stage between 20–29 November 2018. Twenty little penguins were selected randomly from a study site with 100 nests. Breeding penguins were captured from artificial nest boxes, weighed (to the nearest 10 g using a spring balance) and equipped with GPS and accelerometer data-loggers (10 x 25 x 40 mm, 20g, AxyTrek, TechnoSmArt, Italy) attached to their lower back using waterproof tape [13]. After one or two foraging trips, birds were recaptured in the nest, weighed, and the loggers removed before returning to their nests. These devices recorded data at pre-set intervals: tri-axial acceleration at 100 Hz, GPS positions every 10 s and pressure every 1s. Note the devices also recorded the in-situ temperature every second, but the sensor, embedded in resin, has a slow time response that is not sensitive enough to monitor the rapid temperature changes experienced by penguins as they move through the water column (S1 Fig). Although we could not follow the method of [7] and [8] to determine the thermoclines from the on-board temperature measurement, we got daily detailed satellite data on water temperature (see below). Devices were set to record daily between 5 am and 9 pm. Adults were weighed after they have delivered the meal to chicks. The body mass change before and after the foraging trip was used as a proxy for change in the adult body condition [14]. We also used a larger body mass dataset representative of the population recorded automatically by an Automated Penguin Monitoring System (APMS, see details in [5]). The APMS recorded the date-time and body mass (to the nearest g) of arrival and departure from the colony of all penguins that passed the weighbridge. Data were filtered to include weights between 700-1700g. Weights outside this range are considered outliers [14].

The experiment was approved by the Phillip Island Nature Parks Animal Experimentation Ethics Committee (approval number 3.2016) and a research permit number 10008506 from the Department of Environment, Land, Water, Planning Victoria, Australia.

### Depth, acceleration and GPS data processing

After logger recovery, data were downloaded. Pressure and acceleration data were analysed using Igor Pro (Wavemetrics, Version 8.02, Oregon, USA). The pressure in millibars was converted to water depth in meters. A series of metrics were automatically calculated with a purpose-written macro in Igor Pro [15, 16], including dive depth (>1m) and duration. From the acceleration data, we calculated the vectoral dynamic body acceleration (VDBA, [17]), which integrates the acceleration signals over the three spatial axes, representing the whole-body activity. During prey pursuit, VDBA increases when penguins increase flipper beat frequency or amplitude, or fast change in the body angle [18, 19]. Prey encountered and pursuit occurred when the VDBA value was higher than 0.5 g [16]. The first 5 m of the descent phase were excluded from prey encounter analysis as birds beat their flippers vigorously to overcome buoyancy in this phase, giving false positive information of a prey encounter [18, 20]. To investigate prey capture rate for each stage of the storm event, the cumulative prey encounter durations were summed every 15 minutes for each bird and averaged (± SE) them for each stage of the storm event: Before, During and After.

Foraging efficiency was calculated as

$$Foraging\;efficiency=\frac{Total\;number\;of\;dives\;with\;prey\;encounters}{Total\;number\;of\;dives}$$

GPS data were analysed using R [21]. Next, the distance, time and speed between consecutive GPS locations were calculated using the "raster" R package [22]. The total distance covered by trips and the maximum distance from the colony were then calculated. Finally, the time of the spatial location was matched to each dive time using "simecol" R package [23].

Among all the birds, one penguin made a two-day foraging trip and two penguins were sampled for two consecutive one-day trips. We treated each of these days as an independent foraging trip as no difference was found when including only one of the two foraging trips (i.e. first or second one) in the statistical tests (S1 and S2 Tables).

### Environmental parameters

The thermal structure data of the water column were obtained from the Copernicus marine environment monitoring service (http://marine.copernicus.eu/services-portfolio/access-to-products/?option=com_csw&view=details&product_id=GLOBAL_ANALYSIS_FORECAST_PHY_001_024). The daily mixed layer thickness was extracted from the 20^th^ to the 29^th^ of November 2018 from the high resolution (1/12° grid resolution) global analysis and forecasting system PSY4V3R1, using Nemo ocean model [24], "ncdf4" package [25] and "raster" package [22] within the R environment [21]. The mixed layer thickness is defined as the depth at which temperature difference from the surface reaches 0.2°C (the reference depth for the surface is set at 10 m to avoid most of the strong diurnal cycle in the top few meters of the oceans) and is a good indicator of whether the water column is thermally mixed or not [26]. We calculated the mean of the daily mixed layer thickness in the areas used by penguins.

Wind speed was obtained from the coastal weather station located at Rhyll, Phillip Island, Australia (38°27’ S, 145°18’ E, http://www.bom.gov.au, Bureau of Meteorology). We obtained two daily weather measurements (9 am and 3 pm) with an average value calculated for each day. We assessed day length in relation to the penguins’ trip duration from https://www.timeanddate.com/sun/@2153331?month=11&year=2018.

### Statistical analysis

Statistical analyses of dive parameters were performed in R version 3.4.3 [21]. Data exploration was carried out following the protocol described in [27]. All dive parameters were analysed using Generalised Linear Models (GLM) with a Gaussian family and an identity link function. The number of dives, the average of prey encounter events, body mass at departure and return of weighbridge birds were tested separately with a Poisson distribution and a log link function within GLM [28]. A beta distribution with a logistic link function was applied for foraging efficiency by using "*betareg"* R package [29]. Multiple comparisons were processed using Tukey’s post hoc test with "*PMCMRplus"* R package [30]. The variable sex was pooled together as differences between sexes were not significant after statistical testing.

## Results

**Fig 1:**
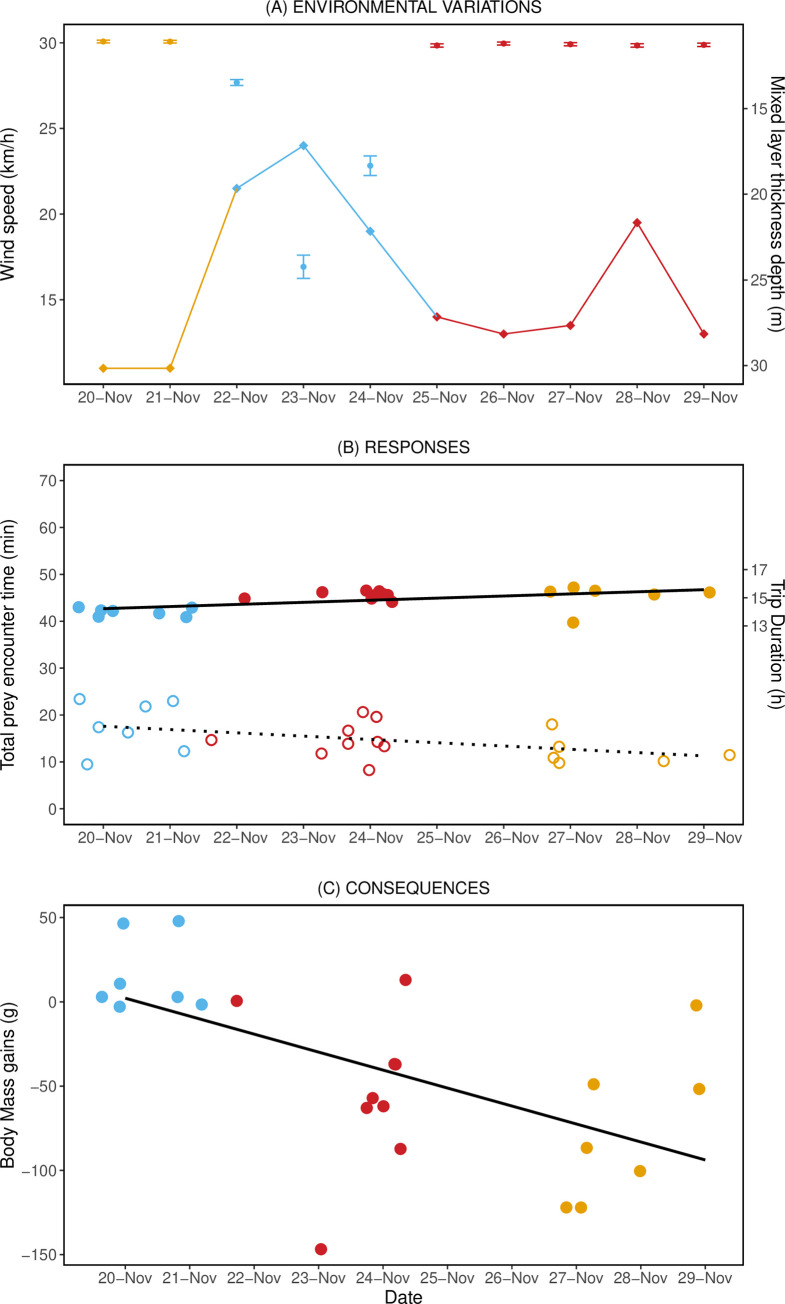
**Comparison of environmental conditions, foraging behaviour and body mass change of little penguins before (blue), during (red) and after the storm (yellow).** A) Daily fluctuation of wind speed (line and diamonds) and mixed layer thickness (dots and error bars, SE). B) Total prey encounter time (empty circles and broken line) and trip duration (full dots and solid line). C) Body mass change over a trip for the instrumented little penguins of the Phillip Island colony. Broken and solid lines correspond to the output of the modelling regressions (see material and methods for details).

During the nine days of study, substantial variations in the wind and thermal structure of the water column were observed. The storm—as indicated by a strong wind of 13 knots—occurred on the 22^nd^ and peaked on the 23^rd^ of November 2018, resulting in mixed waters, i.e. mixed layer thickness increased from 12.5 m to below 23 m (± SE, ± 0.17 to 0.67) between 22^nd^ to 24^th^ of November (Fig 1A). The thermal structure of the water column returned to its initial stratified stage by November 25^th^. This event allowed us to define three stages of the storm event—"Before" (20–21 Nov.), "During" (22–24 Nov.) and "After" (25–29 Nov.) the storm. These stages were used to group and examine the foraging activity of the penguins. The daylength only changed 13.8 minutes over the 9 days of the study and did not influence any of the dive parameters.

**Fig 2:**
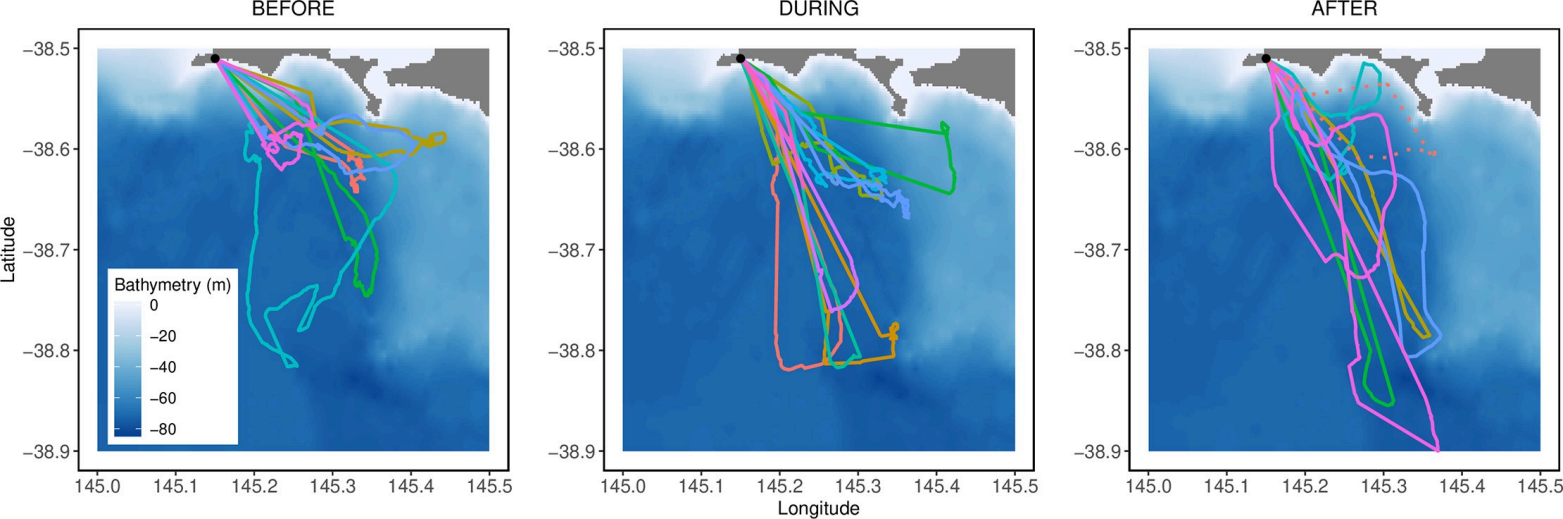
Foraging tracks of little penguins from the Phillip Island colony (black dot) before (from 20^th^ to 21^st^), during (from 22^nd^ to 24^th^) and after (from 27^th^ to 29^th^) a storm event in November 2018. Bathymetry is represented as a blue hue with darker tones indicating deeper waters. The dotted track represents bird #3005.

Birds mainly foraged south-east of their breeding site in all three stages of the storm event with no visual differences in zones visited (Fig 2). The total distance covered by birds and the maximum distance they reached increased during, and especially after the storm but no statistical differences found amongst the three stages (Table 1). One bird (ID #3005, see Table 1) behaved differently (i.e. a substantially higher prey encounter time at shallow depth) compared to all other birds. As such, parameters for this bird are presented separately from the other individuals. Apart from time spent encountering prey, removing this bird from the statistical analysis did not affect on the interpretation of the results (S3 and S4 Tables).

**Table 1:** Comparison of diving parameters of little penguins from Phillip Island, Australia, foraging at guard phase of breeding, before, during and after a storm event in 2018 (see text).

	Before	During	After	Bird #3005
	20–21 Nov	22–24 Nov	25–29 Nov	
Number of individuals [days]	6[7]	8[9]	5[6]	1
Number of dives per day*	484.0 ± 93.8 **a**	631.7 ± 85.3 **b**	567.5 ± 137.7 **c**	1433
Trip duration (h)*	14.0 ± 0.3 **a**	15.2 ± 0.3 **b**	15.1 ± 0.9 **b**	14.4
Maximal distance from the colony (km)	26.3 ± 7.0	27.7 ± 7.3	33.7 ± 11.4	22.1
Total distance covered (km)	68.0 ± 5.7	68.6 ± 15.6	81.2 ± 12.7	53.6
Total time underwater (h)	6.8 ± 1.2	7.3 ± 6.9	7.0 ± 1.4	6.2
Total prey encounter time (min)*	17.7 ± 5.4 **a**	14.8 ± 3.8 **b**	12.3 ± 3.1 **c**	65.3
Prey encounter depth (m)	16.5 ± 4.0	12.6 ± 3.8	14.5 ± 5.5	2.6
Foraging efficiency	0.35 ± 0.09	0.29 ± 0.07	0.28 ± 0.09	0.64
Body mass change (instrumented birds, g)*	15.7 ± 23.7 **a**	-54.4 ± 47.5 **b**	-80.0 ± 46.9 **b**	-50
Number of individuals crossing APMS (departure & return)	82 & 173	184 & 372	278 & 624	
Body mass at departure (APMS, g)*	1143.4 ± 305.2 **a**	1096.6 ± 251.6 **b**	1098.7 ± 302.9 **b**	
Body mass at return (APMS, g)*	1205.8 ± 215.4 **a**	1196.7 ± 271.1 **b**	1199.6 ± 243.8 **b**	

Values are expressed as mean ± SE for the stage of the storm event. Asterisks indicate significant differences in the GLM test (p<0.05). Identical letters indicate no significant differences between stages (Tukey’s post hoc tests, p>0.05). APMS is the acronym for Automated Penguin Monitoring System (cf. Methods). Note bird #3005 is presented separately from the rest of the After sample (see text).

A total of 14,217 dives were analysed over the 9-day period (Table 1). The mean foraging trip duration and the mean number of dives per trip increased significantly in the During and After stages of the storm event (Table 1; Fig 1B). However, the time spent underwater per trip, the depth at which prey were encountered, and the foraging efficiency did not differ among stages (Table 1). Moreover, the prey encounter time differed significantly between all stages, being the highest in the Before stage and lowest in the After stage (Table 1; Fig 1B).

**Fig 3:**
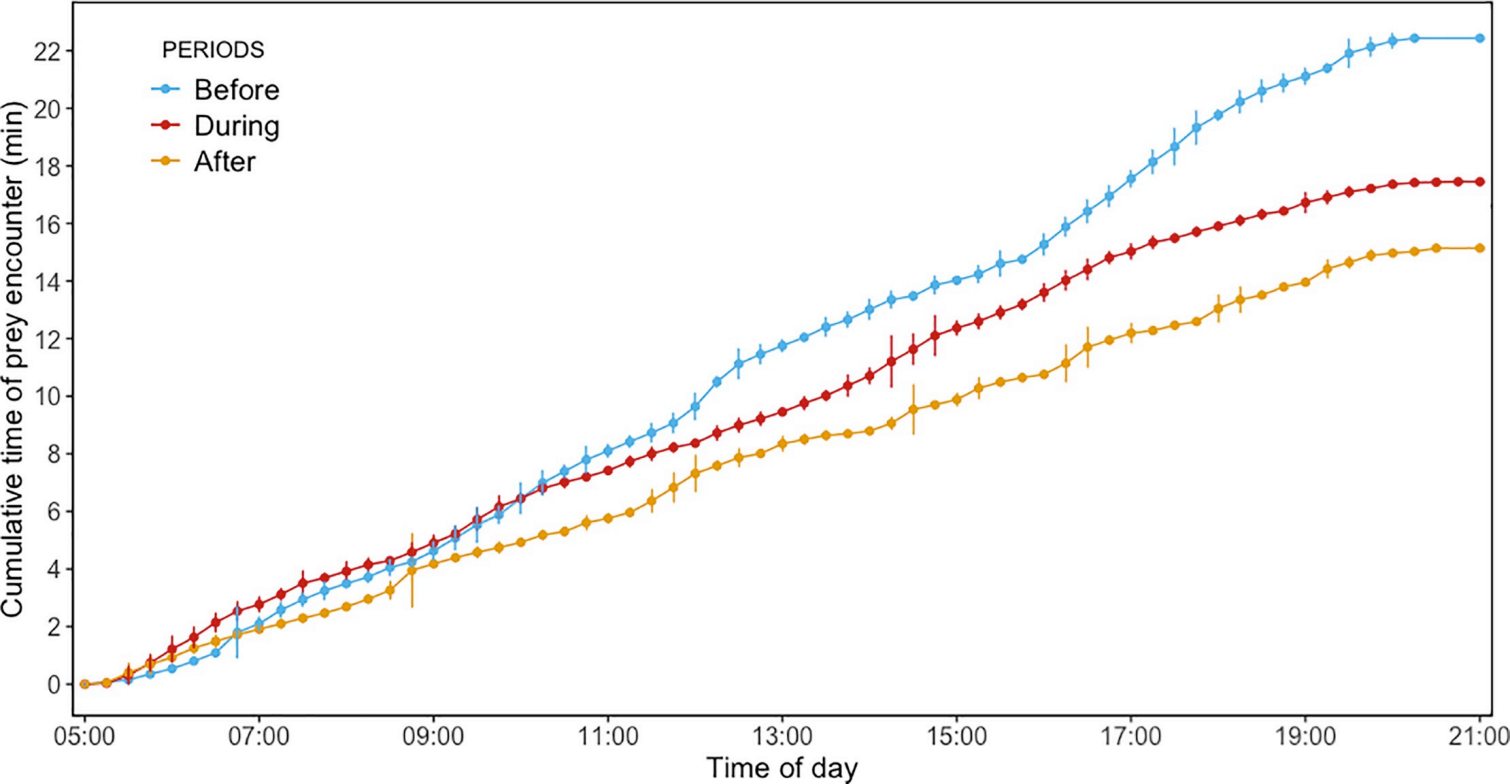
The cumulative time of prey encounter of little penguins as a function of the time of day for the three stages. Blue, red and yellow curves correspond to "Before", "During" and "After" stages, respectively.

Bird #3005 spent the highest total prey encounter time per trip (65.3 min) and had the highest foraging efficiency (0.64, Table 1). Excluding Bird #3005, the cumulative time encountering prey increased faster before than during and after the storm, especially in the second half of the day (Fig 3). In addition, foraging efficiency decreased from Before to both During and After stages, although this was not statistically significant (Table 1).

Finally, the average body mass change measured on our instrumented birds was statistically different between stages (Tukey’s post hoc tests: p-value < 0.001). The body mass change was positive before the storm, becoming negative during and after it (Table 1; Fig 1C). This pattern was consistent with the significant decrease in the mean body masses at departure and return of penguins crossing the weighbridge between before and during/after the storm (Table 1, Tukey’s post hoc tests: p-value < 0.001). Moreover, note that bird #3005 had a much longer prey encounter time and higher foraging efficiency than other birds, but its body mass change was similar to that of other birds from the same stage (Table 1).

## Discussion

Penguins increased their trip duration, had a lower prey encounter rate and decreased body mass over their trips during the storm, with continued adverse effects on the foraging activity several days after the storm ceased.

Adverse weather affects the breeding activity and success of seabirds, either directly or indirectly [31]. For instance, exposure to extreme winds during storms may kill chicks and adults of albatrosses by throwing them on rocks, cliffs; while high waves formed in these storms can cause inundations of penguins’ nests, leading to massive breeding failures [32]. High winds prevent flying birds from accessing their nests or increase the flight cost and decrease the food delivery rate of flying birds [33, 34]. Diving seabirds spend less time foraging under strong wind or high wave conditions [35, 36]. For example, strong winds influence the water masses where birds forage, thereby affecting the foraging success of birds. The negative effect of wind may happen through an increase in the turbidity that can influence the ability of birds to detect and capture prey [37], or due to a mixing of the water column that affects prey behaviour and distribution [31, 38]. The latter is what we suspect happened in our study: the storm changed the thermal structure of the water column; a deepening of the mixed layer being indicative of a deepening or disappearing of the thermocline. Strong wind and changes in the thermal stratification of the water column reduced the ability of little penguins to capture prey efficiently [7–10, 39], as also shown in African penguins (*Spheniscus demersus*) [40]. Birds increased their foraging effort through either a deepening of the dive depth (African penguins) or increased trip duration, a greater diving activity and a shorter time spent encountering prey (little penguins).

All the studies mentioned above examined the immediate impact of the storm. Our study goes further as we were able to monitor the foraging behaviour of birds from the same colony before, during and after a storm event. Indeed, the foraging activity was altered by the storm event that resulted in a reduced body mass gain of adult little penguins among all stages of the storm. Although the perturbation lasted only three days and the thermal structure of the water column returned to its previous state before the storm, the adverse effects on the foraging behaviour of little penguins continued for several more days. The continued reduction in the prey encounter time suggests that prey were still widely dispersed after the physical environment was back to its initial state following the storm. Thus, there may be an additional lag of time for the prey to aggregate and again be available for penguins. This lag is difficult to quantify, but our data suggest that foraging may be still less profitable 3 to 4 days after the storm due to the low prey encounter.

Time spent encountering prey is a proxy of the prey abundance available to the birds. Although this parameter cannot define the extent to which prey encounters were successful, it provides an useful index to compare an animal performance at different time [7, 17–19]. Nonetheless, in addition to the decrease in prey encounter time during the storm, the impacts of the storm on the foraging behaviour of little penguins are also reflected by their longer foraging trips and a greater number of dives. These results are furthermore supported by the negative body mass change of instrumented penguins and lighter body mass of weighbridge penguins.

During guard, parents alternate their foraging each day, and thus, prolonged adverse environmental conditions are likely to affect both parents [5]. Our results suggest that if the foraging success deteriorates for both parents, this will affect the chick growth. Nevertheless, documenting the long term effects of stochastic events, like this storm, is inherently difficult. Thus, we cannot extrapolate from our findings of a period of lower prey encounter and foraging success to possible impacts on chick growth and fledging success. As unfavourable conditions [9] and abrupt changes are likely to occur more frequently in the future [11, 12, 41], an accumulation of events like the one in our study over a breeding season will likely have significant negative consequences on seabird populations [31].

## Supporting information

S1 Fig Depth and temperature during two dives of little penguin recorded by AxyTrek, TechnoSmArt, Italy. **Temperature resolution is 0.1°C and response time is 15s.** Depth rate change of penguins can be up to 2m/s and the maximum temperature difference between descent and ascent phase at the same depth is 0.6°C.
(DOCX)

S1 Table Estimated regression parameters, standard errors (SE), z-values and P-values for the generalized linear models including only one of the two foraging trips (i.e. first or second one). (DOCX)

S2 Table Estimate comparison parameters, confidence intervals and P-values for the Multiple comparison on means results (Tukey’s post hoc test) including only one of the two foraging trips (i.e. first or second one). (DOCX)

S3 Table Estimated regression parameters, standard errors (SE), z-values and P-values for the Generalized Linear Models when including or excluding bird #3005. (DOCX)

S4 Table Estimate comparison parameters, confidence intervals and P-values for the Multiple comparison on means results (Tukey’s post hoc test) when including or excluding bird #3005. (DOCX)
