# Supplementary material for: The consequences of chaos: Foraging activity of a marine predator remains impacted several days after the end of a storm
Source: PLoS One. 2021 Jul 9;16(7):e0254269. doi: 10.1371/journal.pone.0254269 (PMC8270419; doi:10.1371/journal.pone.0254269)
Supplement: S1 Fig — Temperature resolution is 0.1°C and response time is 15s. Depth rate change of penguins can be up to 2m/s and the maximum temperature difference between descent and ascent phase at the same depth is 0.6°C. (DOCX) [file pone.0254269.s001.docx]

**S1 Fig.** Depth and temperature during two dives of little penguin recorded by AxyTrek, TechnoSmArt, Italy. Temperature resolution is 0.1°C and response time is 15s. Depth rate change of penguins can be up to 2m/s and the maximum temperature difference between descent and ascent phase at the same depth is 0.6°C.

**
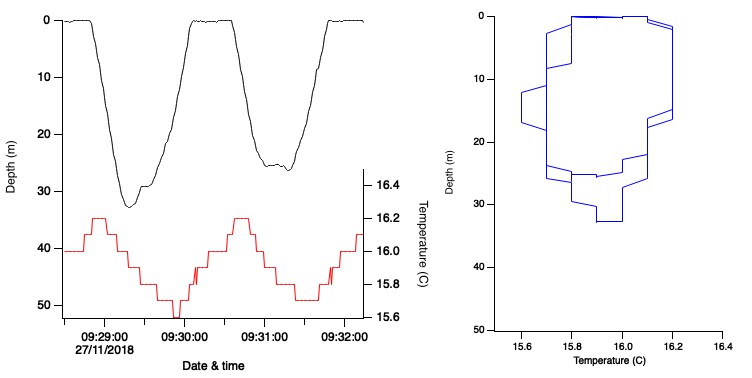
**
