# Supplementary material for: The consequences of chaos: Foraging activity of a marine predator remains impacted several days after the end of a storm
Source: PLoS One. 2021 Jul 9;16(7):e0254269. doi: 10.1371/journal.pone.0254269 (PMC8270419; doi:10.1371/journal.pone.0254269)
Supplement: S1 Table — (DOCX) [file pone.0254269.s002.docx]

**S1 Table.** Estimated regression parameters, standard errors (SE), z-values and P-values for the Generalized Linear Models including only one of the two foraging trips (i.e. first or second one).

|  | | Estimate | | SE | *z value* | | | *P*‐value |
| --- | --- | --- | --- | --- | --- | --- | --- | --- |
| *Number of dives per day* | |  | | | | | | |
| Including first trip (day1) | Intercept | 6.56 | 0.02 | | 428.97 | | < 0.001 | |
|  | Stage.After | -0.45 | 0.02 | | -18.40 | | < 0.001 | |
|  | Stage.During | -0.08 | 0.02 | | -3.92 | | < 0.001 | |
| Including second trip (day2) | Intercept | 6.58 | 0.02 | | 432.89 | | < 0.001 | |
|  | Stage.After | -0.40 | 0.03 | | -15.8 | | < 0.001 | |
|  | Stage.During | -0.13 | 0.02 | | -6.57 | | < 0.001 | |
| *Trip duration* | |  | | | | | | |
| Including first trip (day1) | intercept | 14.90 | | 0.23 | 65.64 | < 0.001 | | |
|  | stage.After | -0.95 | | 0.32 | -2.97 | < 0.01 | | |
|  | stage.During | 0.31 | | 0.30 | 1.04 | > 0.05 | | |
| Including second trip (day2) | intercept | 14.94 | | 0.23 | 64.86 | < 0.001 | | |
|  | stage.After | 0.94 | | 0.34 | -2.75 | < 0.01 | | |
|  | stage.During | 0.23 | | 0.30 | 0.80 | > 0.05 | | |
| *Time spent encountering PE* | |  | | | | | | |
| Including first trip (day1) | intercept | 7.13 | | 0.01 | 619.45 | < 0.001 | | |
|  | stage.After | -0.12 | | 0.02 | -7.14 | < 0.001 | | |
|  | stage.During | -0.34 | | 0.02 | -20.95 | < 0.001 | | |
| Including second trip (day2) | intercept | 7.16 | | 0.01 | 629.03 | < 0.001 | | |
|  | stage.After | -0.15 | | 0.02 | -8.44 | < 0.001 | | |
|  | stage.During | -0.37 | | 0.02 | -23.29 | < 0.001 | | |
| *Body mass changes* | |  | | | | | | |
| Including first trip (day1) | intercept | -0.52 | | 0.31 | -1.64 | > 0.05 | | |
|  | stage.After | 1.53 | | 0.44 | 3.46 | < 0.01 | | |
|  | stage.During | 0.14 | | 0.42 | 0.33 | > 0.05 | | |
| Including second trip (day2) | intercept | -0.55 | | 0.33 | -1.65 | > 0.05 | | |
|  | stage.After | 1.59 | | 0.49 | 3.23 | < 0.01 | | |
|  | stage.During | 0.33 | | 0.43 | 0.45 | > 0.05 | | |
