# Supplementary material for: The consequences of chaos: Foraging activity of a marine predator remains impacted several days after the end of a storm
Source: PLoS One. 2021 Jul 9;16(7):e0254269. doi: 10.1371/journal.pone.0254269 (PMC8270419; doi:10.1371/journal.pone.0254269)
Supplement: S2 Table — (DOCX) [file pone.0254269.s003.docx]

**S2 Table.** Estimate comparison parameters, confidence intervals and P-values for the Multiple comparison on means results (Tukey’s post hoc test) including only one of the two foraging trips (i.e. first or second one).

|  | | Estimate | | IC lower | IC upper | | | *P*‐value |
| --- | --- | --- | --- | --- | --- | --- | --- | --- |
| *Number of dives per day* | |  | | | | | | |
| Including first trip (day1) | Before - After | -0.45 | -0.51 | | -0.39 | | < 0.001 | |
|  | During - After | -0.08 | -0.13 | | 0.03 | | < 0.001 | |
|  | During - Before | 0.37 | 0.31 | | 0.42 | | < 0.001 | |
| Including second trip (day2) | Before - After | -0.40 | -0.46 | | -0.34 | | < 0.001 | |
|  | During - After | -0.13 | 0.13 | | -0.09 | | < 0.001 | |
|  | During - Before | 0.27 | 0.27 | | 0.32 | | < 0.001 | |
| *Trip duration* | |  | | | | | | |
| Including first trip (day1) | Before - After | -0.95 | | -1.70 | -0.20 | < 0.001 | | |
|  | During - After | 0.31 | | -0.39 | 1.02 | > 0.05 | | |
|  | During - Before | 1.27 | | 0.56 | 1.97 | < 0.001 | | |
| Including second trip (day2) | Before - After | -0.94 | | -1.74 | -0.14 | < 0.001 | | |
|  | During - After | 0.24 | | -0.46 | 0.94 | > 0.05 | | |
|  | During - Before | 1.18 | | 0.44 | 1.92 | < 0.001 | | |
| *Time spent encountering PE* | |  | | | | | | |
| Including first trip (day1) | Before - After | -0.11 | | -0.16 | -0.08 | < 0.001 | | |
|  | During - After | -0.34 | | -0.38 | 0.30 | < 0.001 | | |
|  | During - Before | -0.23 | | -0.27 | -0.19 | < 0.001 | | |
| Including second trip (day2) | Before - After | -0.14 | | -0.19 | -0.10 | < 0.001 | | |
|  | During - After | -0.37 | | -0.41 | -0.33 | < 0.001 | | |
|  | During - Before | -0.22 | | -0.26 | -0.18 | < 0.001 | | |
| *Body mass changes* | |  | | | | | | |
| Including first trip (day1) | Before - After | 1.53 | | 0.44 | 3.46 | < 0.01 | | |
|  | During - After | 0.14 | | 0.41 | 0.33 | > 0.05 | | |
|  | During - Before | -1.39 | | 0.42 | -3.36 | < 0.05 | | |
| Including second trip (day2) | Before - After | 1.60 | | 0.44 | 2.75 | < 0.01 | | |
|  | During - After | 0.33 | | -0.68 | 1.33 | > 0.05 | | |
|  | During - Before | -1.26 | | -2.33 | -0.20 | < 0.01 | | |
