# Supplementary material for: The consequences of chaos: Foraging activity of a marine predator remains impacted several days after the end of a storm
Source: PLoS One. 2021 Jul 9;16(7):e0254269. doi: 10.1371/journal.pone.0254269 (PMC8270419; doi:10.1371/journal.pone.0254269)
Supplement: S3 Table — (DOCX) [file pone.0254269.s004.docx]

**S3 Table.** Estimated regression parameters, standard errors (SE), z-values and P-values for the Generalized Linear Models when including or excluding bird #3005.

|  | | **Estimate** | | **SE** | ***z value*** | | | ***P*‐value** |
| --- | --- | --- | --- | --- | --- | --- | --- | --- |
| ***Number of dives per day*** | |  | | | | | | |
| Including #3005 | Intercept | 6.53 | 0.01 | | 454.80 | | < 0.001 | |
|  | Stage.After | -0.35 | 0.02 | | -16.00 | | < 0.001 | |
|  | Stage.During | -0.10 | 0.02 | | -4.60 | | < 0.001 | |
| Excluding #3005 | Intercept | 6.34 | 0.02 | | 370.02 | | < 0.001 | |
|  | Stage.After | -0.16 | 0.02 | | -6.56 | | < 0.001 | |
|  | Stage.During | 0.10 | 0.02 | | 4.90 | | < 0.001 | |
| ***Trip duration*** | |  | | | | | | |
| Including #3005 | intercept | 14.99 | | 0.20 | 74.06 | < 0.001 | | |
|  | stage.After | -1.0 | | 0.29 | -3.45 | < 0.01 | | |
|  | stage.During | 0.20 | | 0.27 | 0.73 | > 0.05 | | |
| Excluding #3005 | intercept | 15.09 | | 0.22 | 69.91 | < 0.001 | | |
|  | stage.After | -1.09 | | 0.29 | -3.70 | < 0.01 | | |
|  | stage.During | 0.10 | | 0.28 | 0.35 | > 0.05 | | |
| ***Time spent encountering PE*** | |  | | | | | | |
| Including #3005 | intercept | 7.08 | | 0.01 | 646.34 | < 0.001 | | |
|  | stage.After | -0.11 | | 0.02 | -7.23 | < 0.001 | | |
|  | stage.During | -0.29 | | 0.02 | -18.76 | < 0.001 | | |
| Excluding #3005 | intercept | 0.23 | | 0.02 | 13.20 | < 0.001 | | |
|  | stage.After | -0.05 | | 0.02 | -2.70 | < 0.05 | | |
|  | stage.During | -0.29 | | 0.02 | -16.81 | < 0.001 | | |
| ***Body mass changes*** | |  | | | | | | |
| Including #3005 | intercept | -0.66 | | 0.28 | -2.35 | < 0.05 | | |
|  | stage.After | 1.67 | | 0.40 | 4.20 | < 0.001 | | |
|  | stage.During | 0.38 | | 0.37 | 1.04 | > 0.05 | | |
| Excluding #3005 | intercept | -0.73 | | 0.30 | -2.43 | < 0.05 | | |
|  | stage.After | 1.71 | | 0.41 | 4.17 | < 0.001 | | |
|  | stage.During | 0.46 | | 0.38 | 1.17 | > 0.05 | | |
