# Supplementary material for: The consequences of chaos: Foraging activity of a marine predator remains impacted several days after the end of a storm
Source: PLoS One. 2021 Jul 9;16(7):e0254269. doi: 10.1371/journal.pone.0254269 (PMC8270419; doi:10.1371/journal.pone.0254269)
Supplement: S4 Table — (DOCX) [file pone.0254269.s005.docx]

**S4 Table.** Estimate comparison parameters, confidence intervals and P-values for the Multiple comparison on means results (Tukey’s post hoc test) when including or excluding bird #3005.

|  | | **Estimate** | | **IC lower** | **IC upper** | | | ***P*‐value** |
| --- | --- | --- | --- | --- | --- | --- | --- | --- |
| ***Number of dives per day*** | |  | | | | | | |
| Including #3005 | Before - After | -0.35 | -0.41 | | -0.30 | | < 0.001 | |
|  | During - After | -0.09 | -0.14 | | -0.04 | | < 0.001 | |
|  | During - Before | 0.26 | 0.21 | | 0.32 | | < 0.001 | |
| Excluding #3005 | Before - After | -0.15 | -0.22 | | -0.10 | | < 0.001 | |
|  | During - After | 0.10 | 0.06 | | 0.15 | | < 0.001 | |
|  | During - Before | 0.26 | 0.21 | | 0.31 | | < 0.001 | |
| ***Trip duration*** | |  | | | | | | |
| Including #3005 | Before - After | -1.09 | | -1.66 | -0.31 | < 0.001 | | |
|  | During - After | 0.20 | | -0.43 | 0.83 | > 0.05 | | |
|  | During - Before | 1.19 | | 0.55 | 1.81 | < 0.001 | | |
| Excluding #3005 | Before - After | -1.09 | | -1.78 | -0.40 | < 0.001 | | |
|  | During - After | 0.10 | | -0.55 | 0.75 | > 0.05 | | |
|  | During - Before | 1.19 | | 0.56 | 1.81 | < 0.001 | | |
| ***Time spent encountering PE*** | |  | | | | | | |
| Including #3005 | Before - After | -0.12 | | -0.15 | -0.09 | < 0.001 | | |
|  | During - After | -0.29 | | -0.33 | -0.26 | < 0.001 | | |
|  | During - Before | -1.18 | | -0.21 | -0.14 | < 0.001 | | |
| Excluding #3005 | Before - After | 0.36 | | 0.32 | 0.41 | < 0.001 | | |
|  | During - After | 0.18 | | 0.14 | 0.23 | < 0.001 | | |
|  | During - Before | -0.18 | | -0.22 | -0.14 | < 0.001 | | |
| ***Body mass changes*** | |  | | | | | | |
| Including #3005 | Before - After | 1.67 | | 0.74 | 2.60 | < 0.05 | | |
|  | During - After | 0.38 | | -0.49 | 1.27 | < 0.001 | | |
|  | During - Before | -1.28 | | -2.16 | -0.40 | > 0.05 | | |
| Excluding #3005 | Before - After | 1.71 | | 0.75 | 2.68 | < 0.001 | | |
|  | During - After | 0.46 | | -0.45 | 1.37 | > 0.05 | | |
|  | During - Before | -1.25 | | -2.13 | -0.38 | < 0.01 | | |
